# Synthetic Ligand-Coated Starch Magnetic Microbeads for Selective Extraction of Food Additive Silicon Dioxide from Commercial Processed Food

**DOI:** 10.3390/nano11020532

**Published:** 2021-02-19

**Authors:** Jun-Hee Lee, Sang-Mook You, Ke Luo, Ji-Su Ko, Ah-Hyun Jo, Young-Rok Kim

**Affiliations:** Department of Food Science and Biotechnology, Institute of Life Sciences and Resources, College of Life Sciences, Kyung Hee University, Yongin 17104, Korea; rebirth425@khu.ac.kr (J.-H.L.); ysmleo1@gmail.com (S.-M.Y.); luoke@ouc.edu.cn (K.L.); rhwltn513@naver.com (J.-S.K.); ted79@naver.com (A.-H.J.)

**Keywords:** silicon dioxide, food additive, nanoscale, magnetic separation, affinity ligand, silaffin

## Abstract

The amorphous form of silicon dioxide has long been regarded as a safe food additive (E551) that is widely used in commercially processed food as an anticaking agent. However, starting with titanium dioxide, there have been growing safety concerns regarding to the use of nanoscale silicon dioxide particles in food as food additives. The size, morphology, and chemical properties of inorganic food materials are important parameters to determine its potential toxicity. Therefore, an effective means of extracting an intact form of SiO_2_ from food without altering the physicochemical property of SiO_2_ particles is of great need to accurately monitor its characteristics. Here, we report on an effective magnetic separation method to extract food additive SiO_2_ from food by utilizing a diatom-originated peptide with a specific affinity to SiO_2_ particles. The affinity-based magnetic separation was found to be specific to SiO_2_ particles over other types of inorganic food additives such as titanium dioxide and zinc oxide. The size and morphology of SiO_2_ were shown to not be affected by the extraction processes. This method was successfully applied to extract and characterize the food additive SiO_2_ from six different types of commercial food.

## 1. Introduction

The amorphous form of silicon dioxide, a synthetic amorphous silica (SAS), is authorized as a food additive (E551) in many countries including the European Union and U.S. [1]. Food additive silicon dioxide has been mostly used as an anticaking agent to prevent the various powdered food ingredients from sticking together or clumping [2]. The E551 has a primary particle size of several tens of nanometers and exists in the form of aggregates that are not separated under normal conditions due to the strong van der Waals interaction and hydrogen bonding [1]. The size of aggregates is of importance in terms of the functionality of E551 as an anticaking agent in food. If the size of the E551 aggregate is smaller than 100 nm, its function as a spacer or anti-caking agent is significantly hindered. Food grade silicon dioxide (SiO_2_) has been known to have very low toxicity with a NOAEL (no observed adverse effect level) of over 2000 mg/kg when ingested orally [3,4]. However, various environmental factors such as heating, pH change, and potential interaction with food ingredients imposed during food processing may cause undesirable alterations in the physicochemical properties of food additive SiO_2_ present in processed food, which may lead to changes in its toxicity [5,6,7,8,9,10,11]. In fact, studies have shown that the particle size is an important parameter that determines the penetrating nature of SiO_2_ in biological tissues, which may also increase its toxicity [10,12]. The growing concerns regarding the potential toxicity of nanoscale food additive SiO_2_ have necessitated the effective means of extracting intrinsic forms of SiO_2_ from a range of processed food for the analysis of its physicochemical properties and safety.

Extraction or separation of nanoscale food additives such as synthetic amorphous silica from food is challenging because food is comprised of a range of complicated matrices including carbohydrates, fats, proteins, and various salts [13]. These food components should be removed for the accurate analysis of inorganic food additives present in processed food. Conventional extraction methods consist of a digestion process that degrades food matrices and a separation process that removes digested food matrices, leaving undigested inorganic food additives behind [14,15]. Digestion of food components is typically carried out with strong acid treatment in combination with heating, and the digested components can be removed by centrifugation or filtration. As a food additive, SiO_2_ amorphous silica is resistant to most inorganic acids including hydrochloric acid, nitric acid, and sulfuric acid. Thus, its constituent particle sizes are not likely to be affected by the acid digestion process. However, acid digestion could bring about a significant impact on the hydrodynamic size characteristics of food additive SiO_2_. According to the Derjaguin-Landau-Verwey-Overbeek (DLVO) theory, the formation of an aggregation or agglomerate of SiO_2_ particles can be accelerated by the acid digestion process [16]. The size of the SiO_2_ agglomerate is determined by the balance between the electrostatic repulsion of the silanol groups present on its surface and the van der Waals interaction among the particles. At the pH lower than 3, the van der Waals interactions become dominant along with the decreased repulsion between silanol groups, leading to the growth in the size of the agglomerates. Although the structure of the silica agglomerates would be restored to the original state by adjusting the pH to neutral after acid digestion, it is also possible to have different size distribution patterns by repeated treatments with strong acids. The harsh digestion reaction accompanied by extended treatment with strong acids and an intense heating process would also affect the morphology and size of the constituent particles, which would distort the actual form of SiO_2_ particles present in food. Therefore, in order to analyze the particle size distribution of food additive silica present in the final food product, an effective means of extracting or recovering the food additive silica from processed food without physical or chemical alteration is of great need. Magnetic separation offers an attractive means of capturing and separating a certain target analyte in intact form by using superparamagnetic particles, of which its surface is functionalized with a target specific ligand [17,18,19]. Antibody is the most widely used ligand in the magnetic separation technique due to its highly specific and strong interaction with the target materials [20]. However, the targets of the antibody are typically proteins or other biological components with a molecular weight over a certain range. It is difficult to produce effective antibodies with a specific affinity to inorganic substances like amorphous silica.

In this study, we employed a part of silaffin as an effective ligand to capture and separate food additive SiO_2_ particles from complicated food matrices. Silaffin was first discovered in *Thalassiosira pseudonana*, where the protein is permanently associated with the cell wall of the diatom made of amorphous hydrated silicon dioxide. A small recombinant peptide (silica binding peptide (SBP), 36-amino acid long) was constructed based on the T8 domain of silaffin, showing a specific affinity to silicon dioxide and expressed as a fusion protein with maltose binding protein (MBP). The MBP domain was used to conjugate the SBP to the surface of starch-based magnetic microbeads (SMMBs) by using the intrinsic affinity of MBP to the glucan moiety of the SMMBs. The capture and recovery efficiency of the magnetic separation system with the specific ligand for the food additive SiO_2_ was evaluated. To verify the specificity of this system, two other types of inorganic food additives, titanium dioxide and zinc oxide, were also tested along with the target food additive SiO_2_. Furthermore, the magnetic separation method developed in this study was successfully applied to extract SiO_2_ particles from commercial foods that are sold in the market for further characterization.

## 2. Materials and Methods

### 2.1. Materials

Commercial food additive SiO_2_ (AEROSIL 200F, food grade) was purchased from Evonik Industries AG (Essen, Germany). Food grade titanium dioxide and zinc oxide was purchased from Tioxide Europe SRL. (Ternate, VA, Italy) and Spectrum Chemical Mfg. Corp. (New Brunswick, NJ, USA), respectively. Bovine serum albumin (BSA), casein sodium salt, maltose monohydrate, triton X-100, Bradford reagent, isopropyl-β-d-thiogalactopyranoside (IPTG), sodium dodecyl sulfate (SDS) and tetramethylethylenediamine (TEMED) were purchased from Sigma-Aldrich (St. Louis, MO, USA). BamHI and XbaI were purchased from New England Biolabs Inc. (Ipswich, MA, USA). Luria-Bertani (LB) broth and T4 lagase were obtained from BD Difco (Franklin Lakes, NJ, USA) and Promega Co (Madison, Wl, USA), respectively. Pfu polymerase and dNTP were purchased from Bioneer Co. (Daejeon, Korea). Waxy maize starch and sucrose were obtained from Yakuri Pure Chemicals (Kyoto, Japan) and Samyang Co. (Gyeonggi, Korea), respectively. Acrylamide solution (30%) was purchased from Bio-Rad Laboratory (Hercules, California, USA). Anodized aluminum oxide membranes (AnodiscTM, pore size 0.2 μm) and Ni-NTA agarose resin were purchased from Whatman (Maidstone, UK) and Qiagen Inc. (Valencia, CA, USA), respectively. Six processed foods were purchased from the local market.

### 2.2. Construction of Expression Vector for Bifunctional Fusion Protein, Maltose Binding Protein-Tagged Silica Binding Peptide (MBP-SBP)

The sequence of silica binding peptide (SBP) was based on the T8 domain of silaffin, which was registered in the National Center for Biotechnology Information (NCBI) GenBank database (Poulsen and Kröger 2004) (Table S1). The DNA fragment encoding the T8 domain of silaffin along with the histidine tag (5X-his) was synthesized by Macrogen (Seoul, Korea), as shown below. 

5’ GGA TCC ATG TCG **AAA** CAA **GGC AAA** ACC GAG ATG **AGC** GTG GCC GAT GCC **AAA** GCC **TCG AAA** GAG **TCG** AGC ATG **CCG TCG** TCG **AAA** GCT GCC AAA ATC TTC **AAA GGC AAA AGC** GGG **AAA** *CAC CAC CAC CAC CAC* TGA TCT AGA 3’.

The sequences encoding certain amino acids including lysine, glycine, serine, and proline, were modified to those (bold letter) having a higher codon usage in the host strain, *E. coli* BL21 (DE3) (Supplementary Materials Figure S1). The synthesized gene was flanked by two restriction enzyme sites, BamHI and XbaI, to clone into the multiple cloning site (MCS) of the expression vector, pMAL-c2x. The restriction enzyme sites were underlined, and the genes encoding the his-tag was written in italic. The SBP gene was digested with BamHI and XbaI, and ligated into the MCS of pMAL-c2x using the T4 ligase. The resulting construct, pMAL-c2x::MBP-SBP, was transferred to *E. coli* BL21(DE3) pLysS by the conventional heat-shock method [21]. The transformed *E. coli* BL21 (DE3) pLysS harboring pMAL-c2x::MBP-SBP was screened by growing in a selective media containing ampicillin to the final concentration of 0.1 mg/mL. The sequence of the cloned MBP-SBP gene was confirmed by sequencing the insert region of expression vector.

### 2.3. Expression and Purification of MBP-SBP

*E. coli* BL21 (DE3) pLysS harboring pMAL-c2x::MBP-SBP was grown at 37 °C with agitation (200 rpm) in LB broth containing ampicillin (0.1 mg/mL) to an optical density of 0.5 at 600 nm. The culture was induced with 1 mM IPTG and grown further at 18 °C with constant shaking at 200 rpm for 24 h. The cells were harvested by centrifugation (7000× *g* for 20 min at 4 °C) and stored at 4 °C until use. The bifunctional fusion protein, MBP-SBP, was purified using a Ni-NTA agarose resin (Qiagen, Valencia, CA, USA) under native conditions according to the manufacturer’s instructions. Protein concentration was determined by the Bradford method with bovine serum albumin (BSA) as a standard. The purity and molecular weight of the protein was analyzed by 10% Sodium Dodecyl Sulfate Polyacrylamide Gel Electrophoresis (SDS-PAGE) and Coomassie brilliant blue staining.

### 2.4. Binding Assay of MBP-SBP for Food Additive SiO_2_

Food additive SiO_2_ (5 mg) was incubated with 1.5 mg of MBP-SBP in 1 mL of PBS with gentle rotation at room temperature for 1 h. The food additive SiO_2_, along with the bound SBP on the surface of SiO_2_ particle, was spun down by centrifugation at 21,000× *g* for 5 min. The amount of unbound protein in the supernatant was quantified by the Bradford assay. The binding efficiency of SBP to the food additive SiO_2_ was calculated by Equation (1):

(1) $${\mathrm{Binding}\;\mathrm{efficiency}}_{\mathrm{SBP}}=\;\frac{{\mathrm{SBP}}_{\mathrm{total}}-{\mathrm{SBP}}_{\mathrm{unbound}}}{{\mathrm{SBP}}_{\mathrm{total}}}\times 100$$

where SBP_total_ is the total mass of SBP that is introduced to the binding solution, and SBP_unbound_ is the mass of SBP in the supernatant after the binding and centrifugation. The binding efficiency of SBP to the food additive TiO_2_ and ZnO particles was evaluated through the same procedure described above to assess the specificity of the binding affinity of SBP to silica particles. 

### 2.5. Preparation and Characterization of Starch Magnetic Microbeads Functionalized with SBP (SBP-MBP@SMMBs)

Starch magnetic microbeads (SMMBs) were prepared by co-crystallization of short-chain glucans (SCGs) obtained by debranching the amylopectins from waxy maize starch and dextran coated iron oxide (Dex@Fe_3_O_4_) nanoparticles (~130 nm in diameter) as described elsewhere [22,23,24]. The Dex@Fe_3_O_4_ was spontaneously incorporated into the starch microbeads during the self-assembly process. The synthesized SMMBs were functionalized with SBP to specifically capture and separate food additive silica from the food samples. One mg of SMMBs were incubated with MBP-SBP (150 µg/mL) in 1 mL PBS at room temperature for 1 h with gentle rotation. The surface functionalized SMMBs with SBPs were collected by external magnetic fields, washed three times with distilled water (DW), and stored at 4 °C until use. The morphology and composition of SBP-MBP@SMMBs were analyzed by high-resolution scanning electron microscopy (HR-SEM, MERLIN Carl Zeiss, Oberkochen, Germany) and field emission transmission electron microscopy (FE-TEM, JEM-2100F, JEOL, USA) equipped with energy dispersive x-ray spectroscopy (EDX) elemental mapping of iron, carbon, and oxygen.

### 2.6. Magnetic Separation of SiO_2_ by SBP-MBP@SMMBs

Commercial food additive SiO_2_ was dispersed in 100 mL DW to a final concentration of 0.1 mg/mL. SBP-MBP@SMMBs were introduced to the suspension to a final concentration of 1 mg/mL and incubated at room temperature for 30 min with gentle rotation. The captured magnetic beads along with the bound SiO_2_ particles were collected to the side of the tube by using a neodymium magnet (50 mm × 5 mm × 25.4 mm), and the supernatant containing unbound SiO_2_ was transferred to a fresh tube for quantification. After washing the collected magnetic complex with DW, the SiO_2_ particles were eluted from the surface of SBP-MBP@SMMBs by introducing maltose to the suspension to a final concentration of 10 mM. The mass of silica was quantified by the molybdenum blue spectrophotometric method [25]. Briefly, 40 μL of KOH (10% *w/v*) was added to 100 μL of the test sample and incubated at 90 °C for 20 min. After centrifugation at 1500× *g* for 15 min, 200 μL of the supernatant was mixed with 100 μL HCl (1 M), 20 μL NH4F (1 M), 80 μL DW, and incubated at room temperature (RT) for 30 min. The sample solution was then mixed with 200 μL boric acid (0.5 M) and incubated at RT for 20 min, followed by another round of incubation for 20 min upon the addition of 100 μL ethanol (99%) and 150 μL ammonium molybdate tetrahydrate (5%). Finally, 50 μL of oxalic acid (0.5 M), tartaric acid (0.5 M), and ascorbic acid (2%, *w/v*), respectively, was added to the sample solution, and incubated at RT for 20 min in the dark. The absorbance of the resulting solution was measured at 812 nm using an UV–Vis spectrophotometer (Optigen POP, Mecasys Co. Ltd., Daejeon, Korea). The mass of SiO_2_ in the test sample was calculated by using a standard curve made with a pure form of food additive SiO_2_. The capture efficiency (%CE) and recovery efficiency (%RE) of the SBP-MBP@SMMBs for SiO_2_ particles were calculated by the following equations:

(2) $$\mathrm{CE}\left(\%\right)=\;1-\frac{M_{\mathrm{initial}}-M_{\mathrm{unbound}}}{M_{\mathrm{initial}}}\times 100$$

where M_initial_ is the initial mass of SiO_2_ in the test sample prior to magnetic separation, and M_unbound_ is the mass of unbound SiO_2_ after the magnetic separation:

(3) $$\mathrm{RE}\left(\%\right)=\;1-\frac{M_{\mathrm{initial}}-M_{\mathrm{released}}}{M_{\mathrm{initial}}}\times 100$$

where M_initial_ is the initial mass of SiO_2_ in test sample prior to magnetic separation, and M_released_ is the mass of released SiO_2_ from the SBP-MBP@SMMBs.

For the extraction of food additive SiO_2_ from processed food, 10 g of the food sample was suspended in 100 mL of DW. For food containing a high content of starch such as potato chips, 1 g of the food sample was suspended in 100 mL DW and incubated at 90 °C for 30 min with gentle rotation to solubilize the solid food. SBP-MBP@SMMBs were introduced to the food suspension to a final concentration of 1 mg/mL and incubated at RT for 30 min with gentle rotation to induce complexation of captured magnetic beads with food additive SiO_2_ in the food sample. The captured magnetic bead–SiO_2_ complexes were collected by using a neodymium magnet and washed three times with DW. The bound SiO_2_ particles were eluted from the magnetic beads by treating the complexes with maltose to a final concentration of 10 mM. The capture magnetic beads were removed from the suspension by an external magnetic field.

### 2.7. Characterization of Separated Silica Nanoparticles

The morphology, size, and composition of food additive SiO_2_ recovered from processed food were analyzed by HR-SEM equipped with EDX elemental mapping of silicon (Si) and oxygen (O). The solution containing recovered SiO_2_ was filtered through an AnodiscTM Filter membrane (diameter of 0.02 μm), followed by drying in a vacuum chamber, and a platinum coating to enhance the image contrast. The particle size distribution of food additive SiO_2_ recovered from processed food was estimated by measuring the diameter of at least 100 particles from HR-SEM images. The hydrodynamic size of recovered SiO_2_ were measured by dynamic light scattering (DLS, Zetasizer Nano ZS90, Malvern Instruments). All measurements were performed at a scattering angle of 90 degrees (*n* = 3).

## 3. Results and Discussion 

### 3.1. Preparation of Silica-Specific Ligand Protein, MBP-SBP

**Figure 1:**
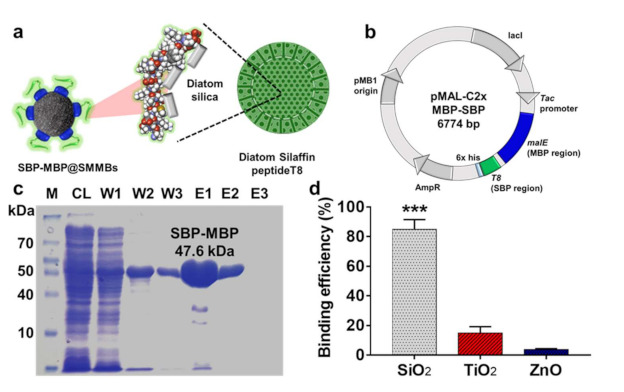
(**a**) Schematic illustration showing the starch magnetic microbeads functionalized with silica binding peptide (SBP-MBP@SMMBs). The SBP was designed based on the T8 domain of silaffin, which exhibits a specific affinity to silica and is associated with the formation of the diatom’s biosilica. (**b**) The map of expression vector, pMAL-C2x, harboring malE and T8 genes, encoding the MBP and SBP in fused form. (**c**) SDS-PAGE analysis of purified MBP-SBP. M, standard size maker; CL, cell lysate; W, washing fraction; E, elution fraction. (**d**) Specific binding efficiency of MBP-SBP for commercial food additive SiO_2_ over other types of inorganic food additive TiO_2_ and ZnO particles. The asterisks (***) indicate significant difference at p < 0.001 for the specific affinity of MBP-SBP for SiO_2_ over the non-target group, TiO_2_, and ZnO. The error bars represent the standard deviation (n = 3).

We employed part of the silaffin from *Thalassiosira pseudonana* as a specific ligand to capture SiO_2_ particles in food. Silaffin was first discovered in the cell wall of a diatom made of amorphous hydrated silicon dioxide and has been shown to remain permanently associated within the silica structure of the diatom (Figure 1a). In particular, the T8 domain consisting of 36-amino acid (residues 165 to 200 of silaffin from *T. pseudonana*) was reported to show sufficient affinity to the silicon dioxide cell walls of the diatom [26,27,28,29,30]. In order to use the silaffin’s T8 domain as a specific ligand to capture SiO_2_ particles, a strand of DNA encoding the T8 domain (Table S1) was chemically synthesized and ligated to the downstream of the malE gene that encodes maltose binding protein (MBP) in expression vector, pMAL-C2x (Figure 1b). The DNA sequence of the T8 domain was partly modified according to the codon usage of the host strain, *E. coli* BL21 (DE3) [31]. The 17 codons in the T8 gene encoding certain amino acids including lysine, glycine, serine, and proline were optimized to high-frequency-usage ones for the effective expression of the ligand protein in *E. coli* (Figure S1). Hereafter, the T8 domain of silaffin will be referred to as silica binding peptide (SBP). The SBP was expressed together with MBP as a bifunctional fusion protein. The MBP domain possessing specific affinity toward maltose or glucan molecules was tagged to the SBP in order to immobilize the SBP to the surface of starch based magnetic microbeads (SMMBs). The constructed expression vector, pMAL-c2x::MBP-SBP, was transferred into *E. coli* BL21 (DE3) for overexpression of the bifunctional ligand protein, MBP-SBP. The ligand protein was successfully expressed from the host strain and its molecular weight was determined to be 47.6 kDa by SDS-PAGE analysis, which is in accordance with the calculated one (Figure 1c). The relatively larger MBP (33.6 kDa) could interfere with the specific binding of SBP (3.8 kDa) to the surface of SiO_2_ particles. To reduce the potential steric hindrance induced by the bulky MBP domain, a 26 amino acid-long linker was introduced between the MBP and SBP.

The purified MBP-SBP was shown to have a specific binding affinity to food additive SiO_2_. When 1.5 mg of MBP-SBP was mixed with 5 mg of food grade SiO_2_ in PBS, over 80% of MBP-SBP was bound to the surface of the silica particles. On the other hand, the ligand protein, MBP-SBP, showed a negligible affinity to the food grade titanium dioxide (TiO_2_) and zinc oxide (ZnO) under the same condition, suggesting that MBP-SBP would be an effective ligand to selectively capture and separate food additive SiO_2_ from a complicated food matrix.

### 3.2. Preparation and Characterization of SBP-MBP@SMMBs

**Figure 2:**
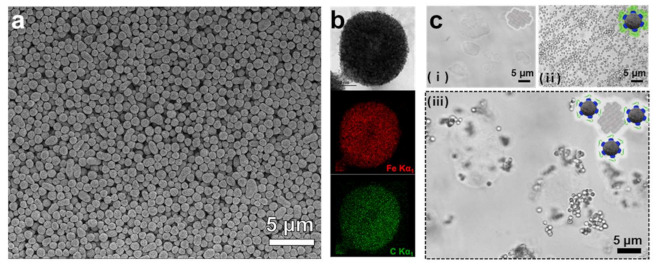
(**a**) Scanning electron microscope (SEM) and (**b**) transmission electron microscopy- energy dispersive x-ray spectroscopy (TEM-EDX) image of SBP-MBP@SMMBs. Elemental iron (Fe) and carbon (C) are represented as red- and green- dots, respectively. (**c**) Transmitted light microscopy of SiO_2_ (i), SBP-MBP@SMMBs (ii) and mixture of SiO_2_ and SMP-MBP@SMMBs that formed an affinity-based complexation (iii) in aqueous solution.

Starch magnetic microbeads (SMMBs) were prepared by the self-assembly process of short chain glucans (SCGs) in aqueous solution [22,23,24,32]. The SCGs obtained by enzymatic debranching of amylopectins from waxy maize starch were induced to crystallize to a spherical form, while dextran coated iron oxide nanoparticles (Dex@Fe_3_O_4_) in the reaction mixture were spontaneously incorporated into the growing starch particles. SEM analysis revealed that well defined and homogeneous SMMBs with a mean diameter of ~750 nm were synthesized by the self-assembly reaction (Figure 2 and Figure S2). According to TEM-EDX analysis, Dex@Fe_3_O_4_ was shown to be well dispersed within the SMMBs, conferring strong superparamagnetic property on the starch beads (Figure 2b and Figure S3). The surface of SMMBs were functionalized with a silica specific ligand by simply mixing 150 µg of MBP-SBP with SMMBs (1 mg) in 1 mL reaction. The conjugation of ligand protein to the surface of SMMBs was solely based on the intrinsic affinity of the MBP domain of the ligand protein to the surface of SMMBs consisting of SCGs. The functionalization process did not require any cross-linking or complicated chemical reaction. The SMMBs functionalized with the specific ligand exhibited a strong affinity to the SiO_2_ in aqueous solution (Figure 2c). Food additive SiO_2_ is typically present in agglomerated form in water. When SBP-MBP@SMMBs were introduced to the suspension containing SiO_2_, the SiO_2_ agglomerates were instantly surrounded by SBP-MBP@SMMBs, which were then separated and concentrated from the solution by an external magnetic field. The results suggest that the SBP domain is well oriented outward on the surface of SMMBs, retaining its specific binding capability for SiO_2_ particles. 

**Figure 3:**
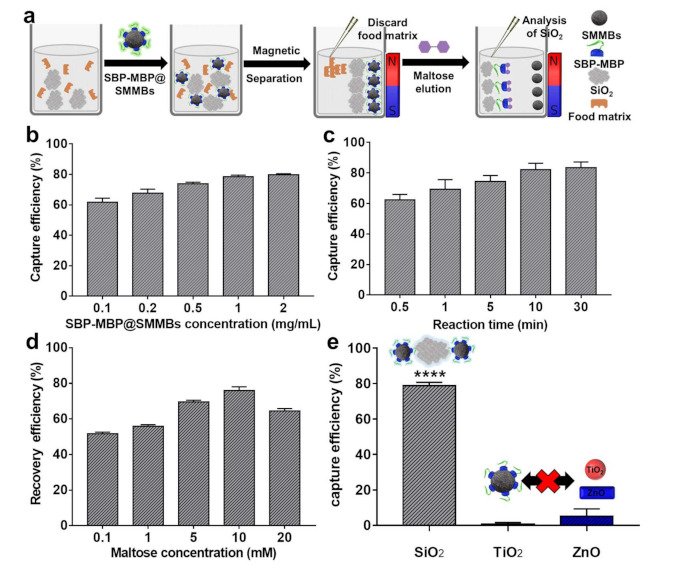
Optimization of experimental condition for the capture and recovery of SiO2 using SBP-MBP@SMMBs. (**a**) Schematic illustration showing the magnetic separation and recovery of SiO_2_ using SBP-MBP@SMMBs. (**b**,**c**) The capture efficiency of SBP-MBP@SMMBs for SiO_2_ as a function of the concentration of SBP-MBP@SMMBs and binding time in aqueous solution. (**d**) The effect of maltose concentration on recovery efficiency of captured SiO_2_ from the surface of SBP-MBP@SMMBs. (**e**) The specificity of SBP-MBP@SMMBs for SiO_2_ over non-target inorganic food additive TiO_2_ and ZnO. The capture test was carried out with 1 mg/mL of SBP-MBP@SMMBs for 10 min. The asterisks (****) indicate significant difference at *p* < 0.0001 for SiO_2_ over the non-target controls such as TiO_2_ and ZnO. The error bars represent the standard deviation of capture and elution efficiency (n = 3). All tests were carried out with 100 ppm of inorganic food additives (SiO_2_, TiO_2_, and ZnO).

The captured SiO_2_ particles on the surface of SBP-MBP@SMMBs were readily released in the presence of free maltose, which competes with SMMPs for the binding sites of MBP (Figure 3a). Excess maltose molecules would saturate all the binding sites of the MBP domain, releasing the ligand protein, MBP-SBP, together with the captured SiO_2_ from the surface of the SMMBs. 

### 3.3. Magnetic Separation of SiO_2_ in the Presence of Food Components

The capability of SBP-MBP@SMMBs for the separation of food additive SiO_2_ from an aqueous medium containing food components was evaluated. First, the reaction conditions for the capture and release of SiO_2_ using SBP-MBP@SMMBs were optimized. For the sample solution containing 100 ppm of SiO_2_, the optimum concentration of SBP-MBP@SMMBs and reaction time for binding were 1 mg/mL and 10 min, respectively (Figure 3b,c). The elution of SiO_2_ from the surface of SBP-MBP@SMMBs was the highest at a maltose concentration of 10 mM (Figure 3d). The SBP-MBP@SMMBs showed high specificity for SiO_2_ with a capture efficiency of around 80% (Figure 3e). The binding of non-target inorganic food additives such as TiO_2_ and ZnO to the captured magnetic particles was negligible with a binding efficiency lower than 10%. Considering that certain processed foods often contain more than two different inorganic food additives, the system proposed in this study would be effective in selectively separating only SiO_2_ particles from food for further analysis. Conventional methods typically require acid digestion, heating, centrifugation, or filtration for the removal of food matrices and the separation of inorganic food additives from food. However, these methods have no means to selectively separate one particular inorganic food additive from another. Therefore, monitoring the physicochemical characteristics of SiO_2_ particles in food that also contain TiO_2_ or ZnO would be difficult with conventional methods.

**Figure 4:**
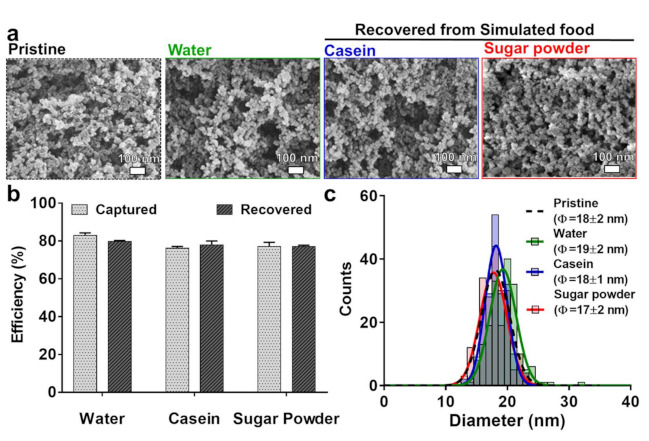
(**a**) SEM images of pristine SiO_2_ as a control and recovered SiO_2_ from simulated food using SBP-MBP@SMMBs. (**b**) Capture and recovery efficiency of SBP-MBP@SMMBs for the SiO_2_ present in various simulated food. (**c**) The size distribution of pristine SiO_2_ (black) and recovered SiO_2_ from simulated food such as water (green), casein (blue), and sugar powder (red). The SiO_2_ content in all simulated foods was 100 ppm.

Separation of SiO_2_ particles from real processed food would be significantly different from the separation of SiO_2_ from a simple aqueous suspension since food is comprised of complicated food matrices such as carbohydrates, proteins, fats, and various salts. Thus, we investigated the capture and recovery efficiency of SBP-MBP@SMMBs for the SiO_2_ that is present in representative food matrices such as casein and sugar powder. As expected, the presence of casein severely affected the recovery of SiO_2_ particles. The casein was speculated to be interfering with the ligand-mediated separation process by coating the surface of SiO_2_ particles. The presence of casein induced the non-specific binding of SiO_2_ to the surface of SBP-MBP@SMMBs, inhibiting the maltose-mediated elution of captured SiO_2_ from the captured magnetic beads. Thus, the recovery efficiency of SiO_2_ from the sample containing casein was shown to be lower than 10% (Figure S4). The inhibitory effect of casein to the recovery of SiO_2_ particles was resolved by introducing a non-ionic surfactant, TX-100, to the binding reaction to a final concentration of 1% (*w/v*). The surfactant was shown to be effective in stripping off the casein from the surface of SiO_2_ particles, restoring the recovery efficiency close to the level obtained in water (Figure 4b). The hydrodynamic size distribution and surface charge of recovered SiO_2_ were not much different from those of the original SiO_2_ that was introduced to the sample containing casein, indicating that the surfactant was readily removed from the surface of SiO_2_ and did not affect the surface property of SiO_2_ during the extraction process (Figures S5 and S6). The separation of SiO_2_ particles with SBP-MBP@SMMBs was also not affected by the presence of sugar powder. SEM analysis also revealed that the size and morphologies of SiO_2_ particles recovered from casein and sugar powder were not much different from those of the original SiO_2_ particles (pristine SiO_2_), suggesting that the effect of the ligand-mediated magnetic separation process on the physical properties of SiO_2_ particles is negligible (Figure 4a,c). In other words, the affinity based magnetic separation was suitable to monitor the physicochemical properties of food additive SiO_2_ in various foods.

### 3.4. Extraction and Characterization of Food Additive SiO_2_ from Commercial Processed Foods

**Figure 5:**
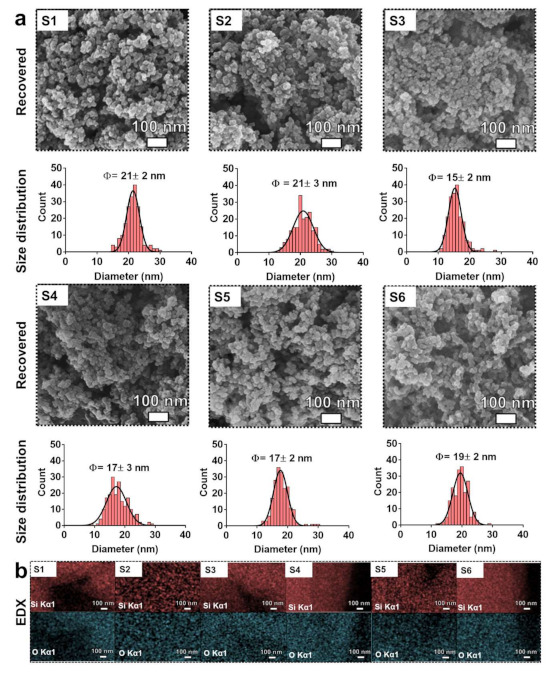
(**a**) SEM images and corresponding size distribution of recovered SiO_2_ using SBP-MBP@SMMBs from commercial processed foods. (**b**) EDX elemental mapping of the corresponding SEM image of (**a**). Elemental silica (Si) and oxygen (O) are represented by red- and cyan-dots, respectively. S1, noodle soup powder; S2, chicken stock powder; S3, Coffee creamer; S4, Coffee mix; S5, Milk tea powder; S6 Potato chip.

The ability of SBP-MBP@SMMBs to extract the food additive SiO_2_ particles from real processed food was evaluated by using six different types of food purchased from the local market. The presence of silicon dioxide was indicated on the ingredient label of all six processed foods. For magnetic separation, 10 g of processed food was suspended in 100 mL of water containing SBP-MBP@SMMBs to a final concentration of 1 mg/mL. For food containing a high ratio of carbohydrate such as potato chips, a heating process (90 °C for 30 min) was employed to solubilize the solid food in water before magnetic separation. Since casein was found to interfere with the ligand-mediated interaction between the SBP and silicon dioxide, food containing a high ratio of casein such as coffee creamer and coffee mix were suspended in water containing a 1% (*w/v*) non-ionic surfactant, TX-100, before magnetic separation. Upon releasing the captured SiO_2_ particles from SBP-MBP@SMMBs by using free maltose, the magnetic beads were removed by a magnet. The recovered SiO_2_ was washed three times with water and dried for further analysis. SEM analysis revealed that the pure form of food additive SiO_2_ was effectively extracted from the six commercial foods (Figure 5).

Most of the background food matrices were eliminated by the specific ligand-mediated magnetic separation, enabling us to monitor the size and morphology of SiO_2_ in food. The extracted SiO_2_ particles were shown as a highly aggregated form with the average diameter of constituent particles of around 15–21 nm. The characteristic morphology and aggregating nature of SiO_2_ particles were also evident in raw (pristine) food additive SiO_2_ particles (Figure 4a). The energy dispersive x-ray spectroscopy (EDX) analysis confirmed that the major composition of the extracted particles was Si and O (Figure 5b). The recovery efficiency of the ligand-mediated magnetic separation for the SiO_2_ particles in processed food was found to be ranging from around 41 to 78% (Table 1). The recovery efficiency was slightly lower in food containing a high ratio of casein, glycerides, and starch, which would interfere with the specific ligand-mediated capturing process. However, the purity of the extracted SiO_2_ particles from all processed foods was high enough for the investigation of the physicochemical characteristics of SiO_2_ particles. The highly specific nature of SBP-MBP@SMMBs for SiO_2_ particles would be particularly attractive for the selective extraction of SiO_2_ from processed food that also contain other types of inorganic food additives such as zinc oxide and titanium dioxide.

## 4. Conclusions

Here, we reported on the ligand-mediated magnetic extraction of food additive SiO_2_ particles from various processed food. The intrinsic nature of silaffin from *Thalassiosira pseudonana*, which has a high affinity toward silicon dioxide was successfully employed as a specific affinity-based ligand to capture and extract food additive SiO_2_ from a processed food sample containing a range of complicated food matrices including fats, carbohydrates, proteins, and various salts. The gene encoding T8 domain of silaffin, silica binding peptide (SBP), was chemically synthesized and ligated to the downstream of the malE gene encoding maltose binding protein (MBP), which was then expressed as a bifunctional fusion protein. The MBP domain of the fusion protein was used as a linker to conjugate the SBP to the surface of starch magnetic microbeads (SMMBs). The SBP-MBP@SMMBs were shown to have a specific affinity for food additive SiO_2_ particles, and its non-specific binding to the other types of inorganic food additives such as titanium dioxide and zinc oxide was negligible. The captured SiO_2_ particles on the surface of SBP-MBP@SMMBs were readily released by free maltose, which competes for the binding sites of the MBP domain with starch-based magnetic microbeads. The size and morphology of the extracted SiO_2_ were not much different from those before magnetic separation, suggesting that the magnetic separation process had minimal effect on the physical properties of the SiO_2_ particles. The ability of SBP-MBP@SMMBs for the extraction of SiO_2_ particles from real food was evaluated by using six different types of commercial processed foods that were labeled to contain the food additive silicon dioxide. The complicated food matrices were effectively eliminated and a pure form of SiO_2_ could be extracted by the specific ligand-mediated magnetic separation method developed in this study. In response to the growing concern of nanoscale food materials, this method would provide an effective and specific means of extracting food additive SiO_2_ particles from processed food to monitor their physical and chemical characteristics.

## Figures and Tables

**Table 1:** The characteristics of SiO_2_ particles extracted from six commercial processed foods.

Commercial Foods	Silica Content (%, *w/w*)	Recovery Efficiency	Constituent Particle Size by SEM	Hydrodynamic Size (nm)	Polydispersity Index
S1 (noodle soup powder)	0.852	77.99	21 ± 2	235.8 ± 60.3	0.477
S2 (chicken stock powder)	0.126	50.52	21 ± 3	292.3 ± 40.2	0.342
S3 (Coffee creamer)	0.119	49.05	15 ± 2	177.8 ± 40.8	0.226
S4 (Coffee mix)	0.042	52.79	17 ± 3	584.3 ± 21.6	0.329
S5 (Milk tea powder)	0.035	44.21	17 ± 2	365.7 ± 21.6	0.338
S6 (Potato chip)	0.254	41.06	19 ± 2	299.9 ± 72.8	0.232

## Data Availability

The data presented in this study are available on request from the corresponding author.

## Supplementary Materials

The following are available online at https://www.mdpi.com/2079-4991/11/2/532/s1, Table S1. Gene sequence of silaffin Sil3 from *T. pseudonana* (NCBI database). Figure S1. DNA sequence of T8 domain of silaffin and SBP. The codons of lysine (K), glycine (G), serine (S) and proline (P) were modified to those having higher codon usage in host strain, *E. coli* BL21 (DE3). Figure S2. Histogram of particle size distribution of SBP-MBP@SMMBs. The diameter of SBP-MBP@SMMBs was estimated by measuring at least 100 particles in SEM images. Figure S3. The magnetic hysteresis of SBP-MBP@SMMBs. The inset shows instant separation of SBP-MBP@SMMBs in aqueous solution under the external magnetic field. Figure S4. The effect of TX-100 treatment on the recovery efficiency of SBP-MBP@SMMBs for the SiO_2_ present in casein-based simulated food. Figure S5. The effect of TX-100 treatment on the hydrodynamic diameter of SiO_2_ before (black) and after (blue) magnetic separation using SBP-MBP@SMMBs from the sample containing casein. Figure S6. The effect of TX-100 treatment on the zeta potential of SiO_2_ before (black) and after (blue) magnetic separation using SBP-MBP@SMMBs from the sample containing casein.
