# Supplementary material for: Synthetic Ligand-Coated Starch Magnetic Microbeads for Selective Extraction of Food Additive Silicon Dioxide from Commercial Processed Food
Source: Nanomaterials (Basel). 2021 Feb 19;11(2):532. doi: 10.3390/nano11020532 (PMC7922398; doi:10.3390/nano11020532)
Supplement: Supplementary file 1 [file nanomaterials-11-00532-s001.pdf]

Supporting information

Synthetic Ligand-Coated Starch Magnetic Microbeads for Selective Extraction of Food Additive Silicon Dioxide from Commercial Processed Food

Jun-Hee Lee†, Sang-Mook You†, Ke Luo, Ji-Su Ko, Ah-Hyun Jo, and Young-Rok Kim*

Institute of Life Sciences and Resources & Department of Food Science and Biotechnology, Kyung Hee University, Yongin, 17104, Republic of Korea

† J.H.L and S.M.Y contributed equally to this work

*Corresponding author.

E-mail address: youngkim@khu.ac.kr

Table S1. Gene sequence of silaffin Sil3 from *T. pseudonana* (NCBI database).

**Gene sequence**
ATGAAGACTTCTGCCATTGCATTGCTTGCCGTTCTCGCCACCACTGCTGCCACCGAGCC
CCGCCGATTGAGAACTCTTGAAGGACATGGGGGAGATCACTCCATCTCCATGTCCATG
CACAGCTCGAAAGCTGAGAAGCAAGCCATCGAGGCAGCTGTTGAGGAGGATGTTGCT
GGCCCTGCAAAGGCAGCCAAGCTTTTCAAGCCCAAAGCAAGCAAGGCTGGTTCCATGC
CTGATGAGGCCGGTGCAAAGAGTGCCAAGATGAGCATGGACACCAAGAGTGGAAAGT
CGGAGGACGCAGCTGCCGTAGATGCCAAAGCTTCCAAGGAATCTCACATGTCTATAAG
TGGTGATATGAGCATGGCCAAGTCACACAAGGCCGAGGCCGAGGACGTCACTGAGAT
GTCCATGGCAAAGGCCGGCAAGGATGAGGCTTCAACCGAGGATATGTGTATGCCCTTC
GCTAAAAGTGACAAGGAAATGAGCGTCAAA**TCGAAGCAAGGAAAGACCGAGATGA**
**GTGTGGCCGATGCCAAGGCCTCAAAGGAGTCTAGCATGCCCTCTTCGAAGGCTGC**
**CAAGATCTTCAAGGGAAAGAGTGGGAAG**TCCGGGAGTCTCTCCATGCTCAAGAGTG
AAAAGGCAAGCTCCGCTCACAGCCTCAGTATGCCAAAAGCTGAGAAGGTCCACTCCAT
GAGCGCTTGA
**Amino acid sequence**
MKTSAIALLAVLATTAATEPRRLRTLEGHGGDHSISMSMHSSKAEKQAIEAAVEEDVAGP
AKAAKLFKPKASKAGSMPDEAGAKSAKMSMDTKSGKSEDAAAVDAKASKESHMSISGD
MSMAKSHKAEAEDVTEMSMAKAGKDEASTEDMCMPFAKSDKEMSVK**SKQGKTEMSVA**
**DAKASKESSMPSSKAAKIFKGKSGK**SGSLSMLKSEKASSAHSLSMPKAEKVHSMSA

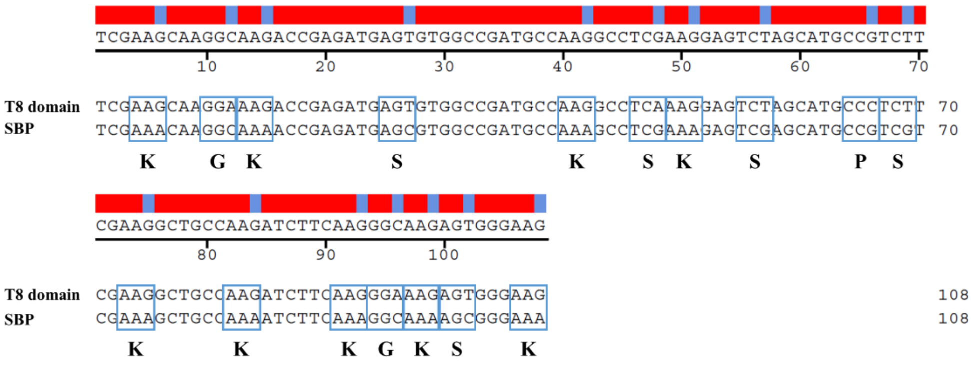

Figure S1. DNA sequence of T8 domain of silaffin and SBP. The codons of lysine (K), glycine (G), serine (S) and proline (P) were modified to those having higher codon usage in host strain, *E. coli* BL21 (DE3).

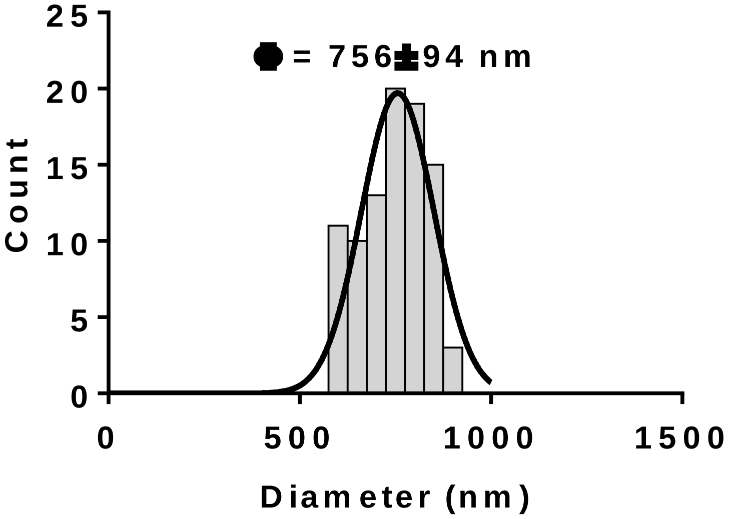

Figure S2. Histogram of particle size distribution of SBP-MBP@SMMBs. The diameter of SBP-MBP@SMMBs was estimated by measuring at least 100 particles in SEM images.

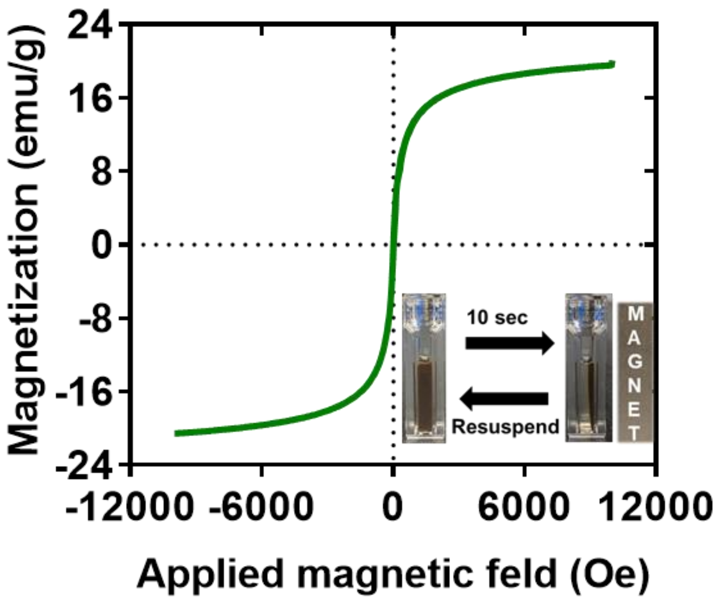

Figure S3. The magnetic hysteresis of SBP-MBP@SMMBs. The inset shows instant separation of SBP-MBP@SMMBs in aqueous solution under the external magnetic field.

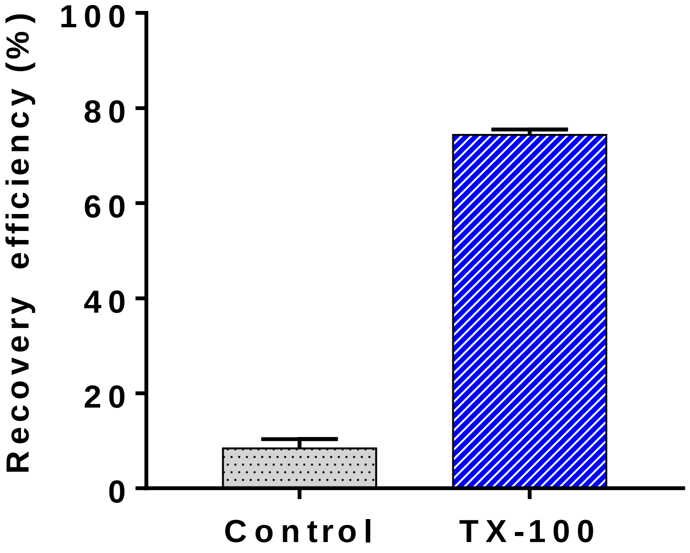

Figure S4. The effect of TX-100 treatment on the recovery efficiency of SBP-MBP@SMMBs for the SiO_2 present in casein-based simulated food.

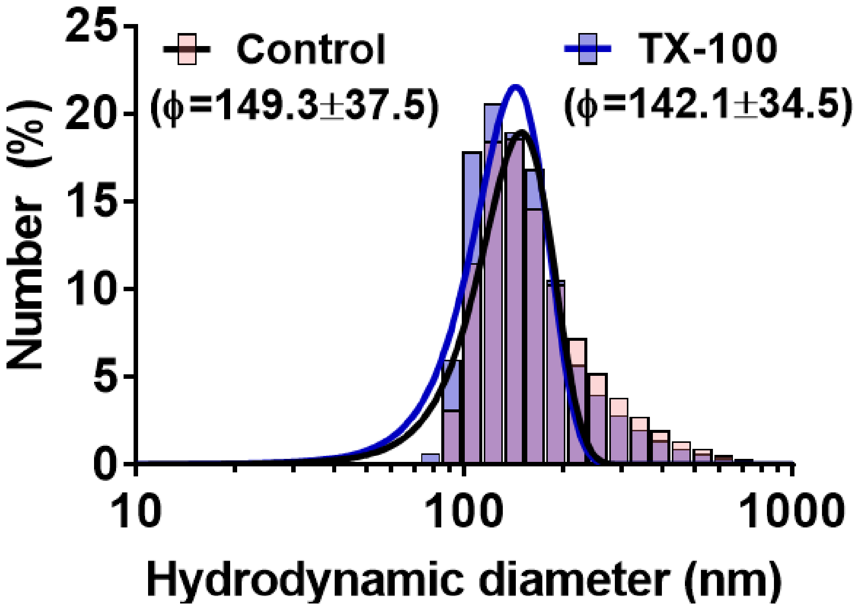

Figure S5. The effect of TX-100 treatment on the hydrodynamic diameter of SiO_2 before (black) and after (blue) magnetic separation using SBP-MBP@SMMBs from the sample containing casein.

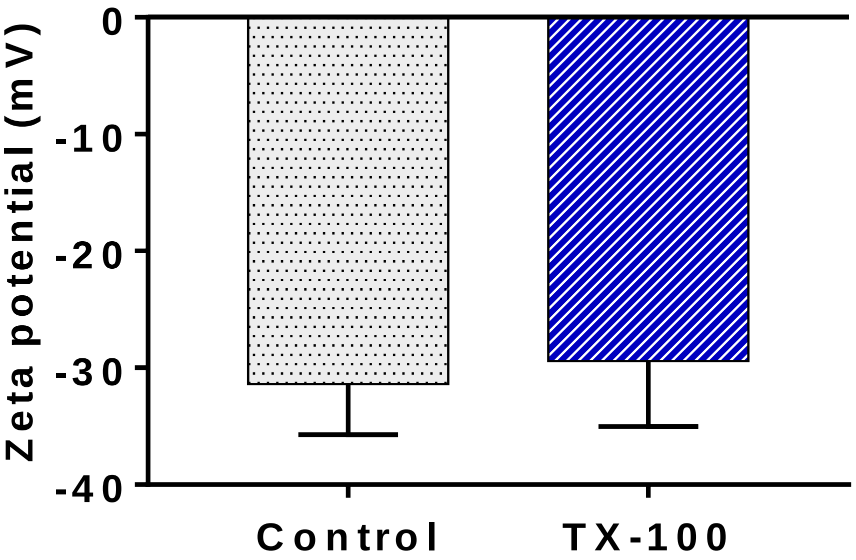

Figure S6. The effect of TX-100 treatment on the zeta potential of SiO_2 before (black) and after (blue) magnetic separation using SBP-MBP@SMMBs from the sample containing casein.
